# Supplementary material for: Advancing Newborn Screening in Washington State: A Novel Multiplexed LC-MS/MS Proteomic Assay for Wilson Disease and Inborn Errors of Immunity
Source: Int J Neonatal Screen. 2025 Jan 10;11(1):6. doi: 10.3390/ijns11010006 (PMC11755445; doi:10.3390/ijns11010006)
Supplement: Supplementary file 1 [file IJNS-11-00006-s001.zip › IJNS-3344349-supplementary.pdf]

Supplementary Information for:

Advancing Newborn Screening in Washington State: A Novel Multiplexed LC-MS/MS Proteomic Assay for Wilson Disease and Inborn Errors of Immunity

Claire Klippel, Jiwoon Park, Sean Sandin, Tara Winstone, Xue Chen, Dennis Orton, Aranjeet Singh, Johnathan Hill, Tareq Shahbal, Emily Hamacher, Brandon Officer, John Thompson, Phi Duong, Tim Grotzer, Si Houn Hahn

Included below:

- Supplementary Figures S1-S2
- Supplementary Tables S1-S6

Supplementary Figures

Figure S1 Linearity curves of six peptides.

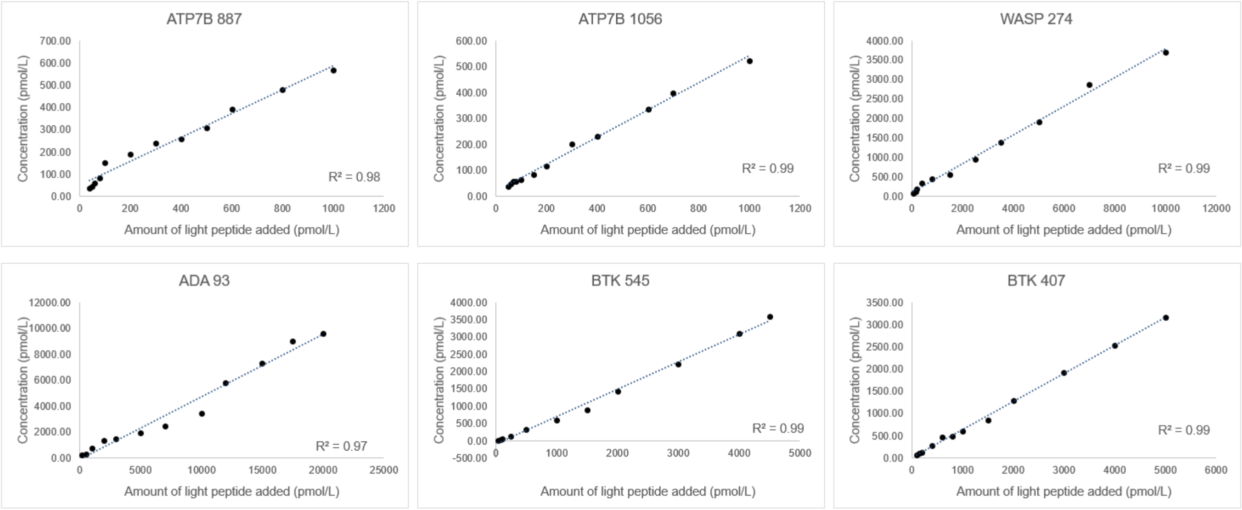

Peptide	Range (pmol/L)
ATP7B 887	59.2-567.9
ATP7B 1056	49.6-525.7
WASP 274	92.2-3709.5
ADA 93	348.7-9647.2
BTK 545	139.5-4065.0
BTK 407	64.7-3166.4

Figure S2. Stability testing results over 12 months. Peptides were processed using kits stored for 0, 3, 6, 9, 12 months after time of receival.

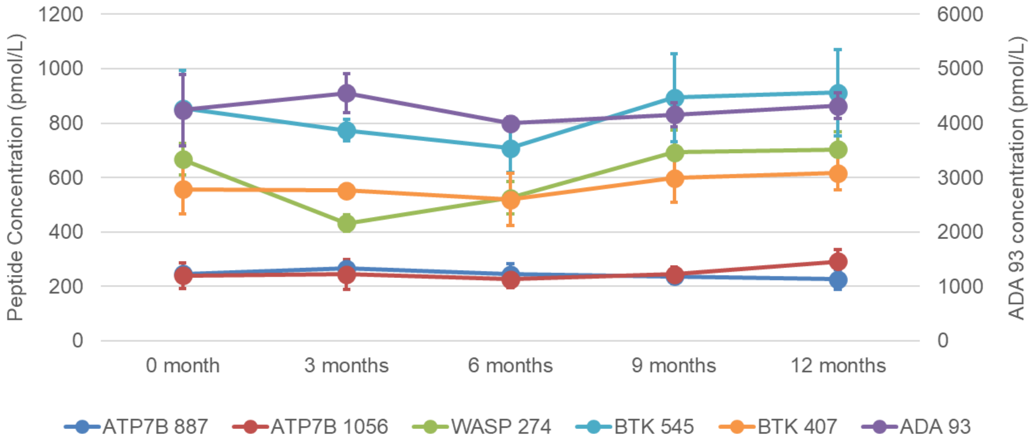

Supplementary Tables

Table S1. Endogenous peptide concentrations (in pmol/L) spiked into fish blood to create each panel member used in the linearity study.

Panel Member	ATP7B 887	ATP7B 1056	WASP 274	ADA 93	BTK 545	BTK 407
Panel 1	40	50	50	200	40	100
Panel 2	50	60	100	500	80	150
Panel 3	60	70	150	1000	100	200
Panel 4	80	80	200	1500	250	400
Panel 5	100	100	400	2000	500	600
Panel 6	200	150	800	3000	1000	800
Panel 7	300	200	1500	4000	1500	1000
Panel 8	400	300	2500	5000	2000	1500
Panel 9	500	400	3500	8000	3000	2000
Panel 10	600	600	5000	10000	4000	3000
Panel 11	800	700	7000	12000	4500	4000
Panel 12	1000	1000	10000	15000	5000	5000

Table S2. Interferent types, concentrations, and control solvents spiked into 100% fish blood and 30% human blood for interference testing.

**Interference**	**Concentration**	**Unit**	**Control Solvent**
Unconjugated Bilirubin	0.4	mg/mL	Diluent B
Conjugated Bilirubin	0.4	mg/mL	Diluent A
Galactose	0.6	mg/mL	Water
Glucose	10	mg/mL	Diluent A
EDTA	3.4	µM	Diluent A
Heparin	3.3	µg/mL	Water
Total Protein	50	mg/mL	Water
Hemoglobin	10	mg/mL	Diluent A
Cholesterol	400	mg/dL	Ethanol
Triglycerides	1500	mg/dL	Ethanol
Gamma-globulin	2.5	g/dL	Water

Table S3. Blank sample concentrations (pmol/L), interferent-spiked sample concentrations (pmol/L), % difference between blank and interferent-spiked samples, and *p*-value of all six target peptides in 30% human blood spiked with 11 types of common interferents. Peptide concentrations below LOQ (lower limit of quantification) were removed and are displayed as N/A.

	Unconjugated Bilirubin				Conjugated Bilirubin				Galactose			
	Blank Sample Concentration (pmol/L)	Interferent Sample Concentration (pmol/L)	% Difference	*p*- value	Blank Sample Concentration (pmol/L)	Interferent Sample Concentration (pmol/L)	% Difference	*p*- value	Blank Sample Concentration (pmol/L)	Interferent Sample Concentration (pmol/L)	% Difference	*p*- value
ATP7B 887	96 ± 19.4	94.6 ± 22.5	-1.4	0.91	89.6 ± 18.5	116.5 ± 58.5	30.0	0.32	84.1 ± 10.5	92.6 ± 22.1	10.1	0.42
ATP7B 1056	94 ± 19.4	92.8 ± 18.4	-1.3	0.92	75.5 ± 7.2	70.7 ± 14.7	-6.4	0.49	74.9 ± 7.6	78.7 ± 5	5.0	0.34
WASP 274	N/A	N/A	N/A	N/A	N/A	N/A	N/A	N/A	N/A	N/A	N/A	N/A
ADA 93	774.3 ± 106.6	724.2 ± 86.4	-6.5	0.39	752.8 ± 135.7	722.4 ± 119.9	-4.0	0.69	730.5 ± 227.3	664.1 ± 73.5	-9.1	0.52
BTK 545	N/A	N/A	N/A	N/A	N/A	N/A	N/A	N/A	N/A	N/A	N/A	N/A
BTK 407	71.6 ± 6.9	78 ± 9.4	8.9	0.22	73.4 ± 7.1	84.5 ± 28.3	15.1	0.39	87.6 ± 8.4	80.9 ± 6.6	-7.7	0.15

	Glucose				EDTA				Heparin			
	Blank Sample Concentration (pmol/L)	Interferent Sample Concentration (pmol/L)	% Difference	*p*- value	Blank Sample Concentration (pmol/L)	Interferent Sample Concentration (pmol/L)	% Difference	*p*- value	Blank Sample Concentration (pmol/L)	Interferent Sample Concentration (pmol/L)	% Difference	*p*- value
ATP7B 887	88.2 ± 10.9	80.7 ± 11.7	-8.5	0.28	93.2 ± 7.9	104.3 ± 24.3	11.8	0.33	86.4 ± 18	73.7 ± 12	-14.8	0.18
ATP7B 1056	83.3 ± 25.1	69.7 ± 15.4	-16.3	0.30	78.4 ± 7.8	72.7 ± 6	-7.2	0.19	76.4 ± 9.7	74.3 ± 21.8	-2.7	0.84
WASP 274	N/A	N/A	N/A	N/A	N/A	N/A	N/A	N/A	N/A	N/A	N/A	N/A
ADA 93	629.5 ± 191.7	661.1 ± 185.2	5.0	0.78	748.2 ± 204.3	651.8 ± 96.8	-12.9	0.33	709.7 ± 137.4	714.5 ± 150.9	0.7	0.95
BTK 545	N/A	N/A	N/A	N/A	N/A	N/A	N/A	N/A	N/A	N/A	N/A	N/A
BTK 407	73.4 ± 6.9	70.6 ± 10.9	-3.9	0.60	74 ± 10.8	83.4 ± 12.7	12.6	0.20	84.8 ± 13.8	84.2 ± 11.5	-0.7	0.93

	Total Protein				Hemoglobin				Gammaglobulin			
	Blank Sample Concentration (pmol/L)	Interferent Sample Concentration (pmol/L)	% Difference	*p*- value	Blank Sample Concentration (pmol/L)	Interferent Sample Concentration (pmol/L)	% Difference	*p*- value	Blank Sample Concentration (pmol/L)	Interferent Sample Concentration (pmol/L)	% Difference	*p*- value
ATP7B 887	67.1 ± 16.8	58.4 ± 10.8	-13.0	0.31	83.1 ± 24.5	94.8 ± 29.9	14.2	0.47	91 ± 19	103.3 ± 18.7	13.6	0.28
ATP7B 1056	65.3 ± 9.9	73 ± 11	11.8	0.25	88.8 ± 24.1	78.6 ± 16.4	-11.5	0.41	82.2 ± 17.4	91.5 ± 18.9	11.4	0.39
WASP 274	N/A	N/A	N/A	N/A	N/A	N/A	N/A	N/A	N/A	N/A	N/A	N/A
ADA 93	609.2 ± 81.3	709.1 ± 76.6	16.4	0.05	718.4 ± 184.1	810.1 ± 151.7	12.8	0.37	679.1 ± 182.3	749 ± 69.2	10.3	0.41
BTK 545	N/A	N/A	N/A	N/A	N/A	N/A	N/A	N/A	N/A	N/A	N/A	N/A
BTK 407	58.5 ± 7.5	66 ± 7.3	12.8	0.11	68.2 ± 10	81.7 ± 36.1	19.8	0.41	81.4 ± 23.3	87.5 ± 14.5	7.5	0.60

	Cholesterol				Triglyceride			
	Blank Sample Concentration (pmol/L)	Interferent Sample Concentration (pmol/L)	% Difference	*p*- value	Blank Sample Concentration (pmol/L)	Interferent Sample Concentration (pmol/L)	% Difference	*p*- value
ATP7B 887	74 ± 17.5	60.2 ± 8.2	-18.6	0.12	75.7 ± 12	89.4 ± 17.3	18.1	0.15
ATP7B 1056	71.1 ± 12.9	67.3 ± 14.8	-5.3	0.70	72.5 ± 12.2	86.8 ± 18.1	19.7	0.16
WASP 274	N/A	N/A	N/A	N/A	N/A	N/A	N/A	N/A
ADA 93	863.2 ± 66.1	705 ± 82	-18.3	0.00	604.2 ± 89	700.7 ± 119.4	16.0	0.15
BTK 545	122 ± 4.6	N/A	N/A	N/A	N/A	N/A	N/A	N/A
BTK 407	83 ± 12.9	82.9 ± 15.6	-0.1	0.99	81.2 ± 11.4	77.3 ± 14.3	-4.9	0.62

Table S4. Average concentrations (pmol/L), within-day, between-day and between-user precision (%) across three sites for each peptide and each precision panel member. Peptide concentrations below LOQ (lower limit of quantification) were removed and are displayed as N/A.

		Site 1				Site 2				Site 3			
Peptide	Panel	Concentration	Within-day CV	Between-day CV	Between-user CV	Concentration	Within-day CV	Between-day CV	Between-user CV	Concentration	Within-day CV	Between-day CV	Between-user CV
ATP7B 887	0% NHC	NA	NA	NA	NA	94.8	48.0	63.5	68.8	71.3	36.8	37.8	38.1
	20% NHC	56.8	9.7	11.2	10.6	66.7	14.9	23.6	22.1	92.4	33.0	35.6	37.3
	40% NHC	101.0	13.9	13.8	14.1	109.9	17.0	20.4	20.8	123.7	24.9	26.9	28.1
	60% NHC	154.6	8.3	10.9	10.6	160.8	10.5	11.2	12.8	156.9	20.8	25.5	25.9
	80% NHC	200.5	8.8	11.1	10.6	184.8	7.7	9.5	10.2	197.0	21.5	22.5	21.8
	100% NHC	246.7	11.1	11.0	10.9	248.0	13.5	12.5	12.8	260.6	20.6	23.3	22.3
ATP7B 1056	0% NHC	NA	NA	NA	NA	NA	NA	NA	NA	68.0	NA	NA	NA
	20% NHC	56.0	6.7	6.3	6.2	59.1	5.1	8.5	7.9	63.1	18.9	19.2	18.5
	40% NHC	99.7	11.3	12.1	12.0	79.2	11.8	12.9	12.3	80.9	28.0	28.4	27.7
	60% NHC	168.9	9.9	13.8	13.4	117.5	9.9	11.3	10.9	128.4	23.1	25.2	25.9
	80% NHC	230.9	9.7	12.1	11.8	156.0	6.0	9.7	9.5	178.6	21.2	24.4	23.4
	100% NHC	312.2	12.3	13.6	12.9	223.0	9.3	14.6	13.5	259.3	24.7	26.7	25.5
WASP 274	0% NHC	NA	NA	NA	NA	190.1	19.3	21.8	20.8	83.5	50.4	48.3	50.3
	20% NHC	126.1	23.9	21.9	21.6	188.7	7.4	34.9	31.4	114.8	30.1	40.7	38.0
	40% NHC	217.3	20.3	21.0	20.5	226.1	9.8	35.3	31.8	181.8	32.0	33.6	32.2
	60% NHC	297.7	14.0	15.8	15.0	279.9	11.0	26.0	24.0	244.4	21.8	22.6	23.5
	80% NHC	397.2	15.6	15.2	14.8	324.5	10.8	26.5	24.1	321.2	22.6	26.9	26.1
	100% NHC	559.7	17.2	16.6	16.7	380.9	10.7	17.1	15.9	402.7	24.8	26.2	25.7
ADA 93	0% NHC	337.9	13.6	57.6	55.4	379.6	26.8	24.2	35.2	391.3	31.7	37.5	47.6
	20% NHC	828.6	7.4	11.5	10.8	900.8	6.1	11.2	10.4	1066.2	22.0	36.0	33.7
	40% NHC	1387.8	8.2	9.7	9.2	1390.1	7.6	11.8	11.2	1501.3	21.7	25.2	24.0
	60% NHC	2180.1	5.7	9.0	10.0	2263.5	20.8	24.4	24.4	2224.6	18.7	24.0	25.1
	80% NHC	2854.8	6.5	9.3	9.5	2587.5	10.4	16.1	15.0	2920.6	15.9	20.9	23.8
	100% NHC	3780.2	9.9	11.0	10.5	3324.0	8.2	14.8	14.0	4178.2	20.2	31.0	34.6
BTK 545	0% NHC	NA	NA	NA	NA	NA	NA	NA	NA	166.0	NA	NA	NA
	20% NHC	145.5	21.1	21.0	21.6	152.9	3.3	31.7	32.9	153.3	19.5	22.6	21.5
	40% NHC	299.8	20.0	20.3	19.7	238.4	46.8	41.9	42.3	230.5	38.5	42.4	47.1
	60% NHC	497.1	15.7	16.9	16.7	384.8	13.2	16.3	15.4	360.0	30.6	32.2	31.4
	80% NHC	728.9	28.7	26.9	30.5	533.0	24.3	24.8	25.0	528.1	23.0	24.9	25.8
	100% NHC	865.2	22.7	21.4	20.9	707.4	14.5	13.1	13.4	670.5	24.7	24.3	23.5
BTK 407	0% NHC	NA	NA	NA	NA	53.9	6.8	10.3	10.1	85.9	38.1	49.2	55.0
	20% NHC	141.8	26.2	27.1	26.5	107.3	21.7	23.5	25.5	101.7	38.1	51.7	49.4
	40% NHC	282.3	15.8	16.2	15.7	192.3	34.5	35.2	35.9	191.5	39.9	41.2	40.6
	60% NHC	441.9	11.2	12.4	12.7	268.2	12.5	17.5	18.9	276.8	21.6	23.8	23.6
	80% NHC	616.0	27.3	24.7	30.0	335.3	21.6	21.8	21.8	371.4	21.9	24.7	23.6
	100% NHC	716.3	24.3	22.6	22.2	405.0	11.6	10.9	11.0	482.2	21.3	22.9	23.0

Table S5. Clinical Validation Study Result. 49 genetically confirmed patient samples blinded into clinical validation study at 3 sites are listed. Site 1: Alberta Precision Lab, Calgary, Canada; Site 2: Seattle Children Research Institute, Seattle, WA; Site 3: Key Proteo, Seattle, WA. For each patient, their age, sex, concentration of all six biomarker peptides, clinical and assay diagnosis results, and DNA sequencing results are shown.

				Concentration (pmol/L)						Diagnosis		Sequencing results	
Site	Case	Sex	Age	ATP7B 1056 (<67.5)	ATP7B 887 (<66.6)	BTK 407 (<156.4)	BTK 545 (<112.2)	WASP 274 (<226.4)	ADA 93 (<1145.6)	Clinical Diagnosis	Assay Diagnosis	Variant 1	Variant 2
	1	M	33	3.7	22.4	350.2	431.5	516.7	2834.5	WD	WD	p.R778L	p.Ile582ArgfsTer25
	2*	M	6	0.1	1.5	529.4	689.7	99.4	5417.5	WD	WD/WAS	p.H1069Q	p.Q1372X
	3	F	45	5.4	21.6	226.1	251.9	236.7	1997.4	WD	WD	p.R778L	p.R778L
	4**	M	20	8.5	9.0	547.6	777.7	162.7	5052.8	WD	WD/WAS	p.H628R-fs	c.2304dupC
	5	NA	32	4.8	47.9	443.6	472.7	453.5	3472.3	WD	WD	c.2463delC	c.2463delC
	6	F	22	3.8	13.8	446.1	593.5	606.8	3078.2	WD	WD	p.H1069Q	p.A1135Q-fs*13
	7	F	26	3.6	16.9	238.8	336.7	555.2	3928.2	WD	WD	p.M769H-fs	p.R816S
1	8	F	12	1.9	13.6	142.2	182.9	149.3	2511.6	WD	WD	p.H1069Q	IVS19-1C>G
	9	NA	38	5.9	15.3	492.9	786.5	712.4	4170.3	WD	WD	p.R919W	p.T993M
	10	M	21	7.0	18.5	485.0	817.4	644.3	4267.9	WD	WD	p.R816S	p.Met769HisfsTer26
	11	M	NA	149.6	176.5	4.6	3.0	1090.5	4115.3	XLA	XLA	p.M630K	
	12	M	NA	266.1	261.3	11.7	4.4	1504.6	4595.3	XLA	XLA	p.N530Tfs26*	
	13	M	NA	229.0	251.5	195.4	260.0	53.7	6224.3	WAS	WAS	p.V75M	
	14	M	NB	255.7	332.9	456.6	787.7	28.1	4814.7	WAS	WAS	p.G337Vfs*108	
	15	NA	NA	289.6	272.0	564.9	770.8	642.7	276.2	ADA	ADA	p.R156H	c.800delG
	1	M	15	75.1	18.0	432.8	421.0	695.1	1912.9	WD	WD	p.H1069Q	p.E1064A
	2	M	39	33.3	30.4	698.0	753.4	940.9	3991.2	WD	WD	p.H1069Q	p.H1069Q
	3	NA	9	35.6	17.6	725.8	633.0	1008.2	4649.5	WD	WD	p.H1069Q	p.R919W
	4	F	24	20.8	19.7	586.9	517.7	477.1	4275.0	WD	WD	p.H1069Q	c.2865+1g>a
	5	M	13	26.2	10.6	662.0	643.8	779.1	2367.4	WD	WD	p.R1041W	p.W1353*
	6	F	10	23.7	7.2	927.4	1224.6	956.7	986.7	WD	WD	p.V845S-fs	p.V845S-fs
	7	F	40	23.9	11.2	582.0	735.2	444.5	3660.4	WD	WD	p.M769H-fs	p.K1028S-fs
2	8	F	56	36.9	16.6	419.3	283.8	630.7	4973.8	WD	WD	p.R778L	p.A874V
	9	M	NA	328.9	244.6	32.2	17.1	1902.5	3589.1	XLA	XLA	p.L647P	
	10	M	NA	185.3	176.6	0.0	5.8	2642.2	3518.8	XLA	XLA	p.N530fsX555	
	11	M	NA	286.6	239.3	56.9	25.6	1551.4	4074.2	XLA	XLA	pP619A	
	12	M	NA	100.4	92.6	600.5	660.7	159.2	2677.0	WAS	WAS	p.W252*	
	13	M	NA	177.1	114.3	601.1	463.6	114.0	4687.3	WAS	WAS	p.R211*	
	14	M	NB	354.7	233.6	717.6	809.4	1235.2	660.3	ADA	ADA	c.955-959delGAAGA	3.25 Kb Del
	1	M	8	7.1	19.9	385.7	521.3	381.0	2796.6	WD	WD	p.H1069Q	2304dupC
	2	F	NA	25.2	81.7	678.3	883.4	1557.2	5164.2	WD	WD	p.I1336V	p.C709T
	3	F	13	35.0	57.7	173.2	244.5	95.3	6416.8	WD	WD	p.W939C	p.L1057P
	4	M	6	8.6	16.0	407.8	366.2	105.8	3810.4	WD	WD	p.H1069Q	p.G1011X
	5	F	24	27.1	55.1	441.2	423.2	601.4	5184.1	WD	WD	p.H1069Q	p.Q447L-fs
	6	M	37	40.9	228.3	203.4	176.5	276.1	3780.2	WD	WD	p.H1069Q	p.Met769HisfsTer26
	7	M	NB	57.6	105.3	664.8	832.4	2180.1	6423.0	WD	WD	p.R778L	p.V1106I
	8	F	6	33.1	50.8	473.1	801.5	249.7	3877.3	WD	WD	p.H1069Q	p.G1341D
	9	M	59	14.6	47.5	185.5	279.5	300.6	2264.4	WD	WD	p.L1088X	p.A1135Q-fs*13
	10	M	22	82.6	64.9	344.6	632.0	263.9	3888.3	WD	WD	p.H1069Q	p.W939C
3	11	NA	23	48.1	85.1	474.4	890.8	760.9	4395.6	WD	WD	p.H1069Q	c.2304-2305insC
	12	M	21	19.1	45.6	490.4	716.4	705.7	3701.1	WD	WD	p.R778L	c.1543 +1G>T
	13	F	8	58.9	63.3	463.5	722.2	135.9	4172.2	WD	WD	p.D765Y	p.R1041W
	14	M	22	24.9	41.5	290.3	448.7	596.6	3493.4	WD	WD	p.H1069Q	p. Thr933Pro
	15	M	NA	245.1	223.7	102.5	86.7	1148.9	8240.5	XLA	XLA	p.Arg525Gln	
	16	M	NB	335.2	417.7	52.4	12.4	3594.8	4986.0	XLA	XLA	Based on Family History	
	17	M	NA	98.5	82.4	37.5	25.5	1743.4	3048.2	XLA	XLA	p.M630K	
	18	M	NB	274.6	368.7	528.1	490.4	186.4	5927.1	WAS	WAS	p.D296Vfs*	
	19	M	NA	117.7	123.8	411.6	589.0	223.0	5432.5	WAS	WAS	c.1453+2T>A	
	20	F	NB	296.6	470.2	930.1	1132.9	1950.0	907.1	ADA	ADA	p.L107P	p.L107P

*c.133-38C>A (VUS) in *WAS* gene

** DNA insufficient to sequence

Table S6. Results of the False Negative Study for Clinical Validation for (A) *ATP7B* gene (B) *BTK* gene (C) *WAS* gene (D) *ADA* gene. Site 1: Alberta Precision Lab, Calgary, Canada; Site 2: Seattle Children Research Institute, Seattle, WA; Site 3: Key Proteo, Seattle, WA. For each sample, the sex, concentration of each peptide, DNA sequencing results, and clinical diagnosis are shown.

(A) *ATP7B* gene

Case	Gender	Concentration (pmol/L)		Sequencing	Conclusion
		ATP7B 887	ATP7B 1056		
1	M	126.8	124.4	c.-388C>T	One VUS. No second variant
2	F	103.3	135.1	No variants detected	Negative
3	M	259.9	151.2	No variants detected	Negative
4	M	418.5	190.8	No variants detected	Negative
5	M	114.7	109.2	No variants detected	Negative
6	M	266.8	76.8	c. 447_452delinsC	Likely pathogenic. No second variant
7	M	104.7	72.8	p.His1069Gln	Pathogenic. No second variant
8	F	489.1	122.0	No variants detected	Negative
9	F	149.6	128.8	No variants detected	Negative
10	M	401.1	133.1	p.Asn581Tyr	One VUS. No second variant
11	M	128.0	187.7	No variants detected	Negative
12	M	159.9	137.8	p.Arg1319Gln/p.Leu1015=	p.Leu1015= is benign. One VUS
13	M	197.1	160.5	p.Lys838Glu/p.Gly1186Asp	p.Lys838Glu = pathogenic. One VUS
14	M	130.2	130.9	No variants detected	Negative
15	M	157.9	192.2	No variants detected	Negative
16	F	89.8	128.7	No variants detected	Negative
17	F	180.0	162.9	p.Pro1379Ser*	Benign. No second variant
18	F	126.2	165.5	No variants detected	Negative
19	M	108.0	151.1	No variants detected	Negative
20	M	114.1	151.1	c.2731-2A>G	Pathogenic variant. No second variant
21	M	146.0	198.2	c.1285+5G>T	One VUS. No second variant
22	F	291.8	252.1	No variants detected	Negative
23	F	112.4	118.6	p.Met1169Val	One VUS. No second variant
24	M	200.4	141.6	No variants detected	Negative
25	M	102.4	80.7	No variants detected	Negative
26	F	237.5	176.0	No variants detected	Negative
27	F	156.9	209.9	No variants detected	Negative
28	M	169.3	153.9	No variants detected	Negative
29	M	142.7	177.6	No variants detected	Negative
30	M	142.6	250.1	No variants detected	Negative
31	M	98.5	65.8	p.Arg1319Ter/p.Pro1379Ser	One pathogenic and one benign
32	F	149.3	220.5	No variants detected	Negative
33	F	179.8	280.4	p.Ala1195Thr	One VUS. No second variant
34	M	156.7	212.4	No variants detected	Negative

(B) *BTK* gene

Case	Gender	Concentration (pmol/L)		Sequencing	Conclusion
		BTK 545	BTK 407		
35	M	1032.1	782.2	p.Ile443Thr	Hemizygous for one VUS
36	M	1099.7	696.0	No variants detected	Negative
37	M	1153.7	809.5	No variants detected	Negative
38	M	1235.0	772.2	No variants detected	Negative
39	M	818.9	582.2	No variants detected	Negative
40	M	1171.2	644.8	No variants detected	Negative
41	M	745.0	368.9	No variants detected	Negative
42	M	804.0	513.1	No variants detected	Negative
43	M	964.1	593.1	No variants detected	Negative
44	M	765.3	475.7	No variants detected	Negative
45	M	1099.4	644.3	No variants detected	Negative
46	M	937.4	687.9	No variants detected	Negative
47	M	1329.4	1129.3	No variants detected	Negative
48	M	1125.3	1139.8	No variants detected	Negative
49	M	953.8	1019.0	No variants detected	Negative
50	M	1361.7	1039.8	No variants detected	Negative
51	M	1280.5	1058.2	No variants detected	Negative
52	M	986.1	1150.9	No variants detected	Negative
53	M	985.6	924.6	No variants detected	Negative
54	M	999.7	688.4	No variants detected	Negative
55	M	1005.8	874.1	No variants detected	Negative
56	M	777.7	547.6	No variants detected	Negative
57	M	1336.7	939.7	No variants detected	Negative
58	M	666.9	828.8	No variants detected	Negative
59	M	745.0	368.9	No variants detected	Negative
60	M	1038.1	648.1	No variants detected	Negative
61	M	964.1	593.1	No variants detected	Negative
62	M	765.3	475.7	No variants detected	Negative
63	M	1148.6	1237.3	No variants detected	Negative
64	M	1010.8	1041.8	No variants detected	Negative
65	M	1155.6	1219.1	No variants detected	Negative
66	M	1124.8	1133.8	No variants detected	Negative

(C) *WAS* gene

Case	Gender	Concentration (pmol/L)	Sequencing	Conclusion
		WASP 274		
67	M	1717.7	No variants detected	Negative
68	M	1635.2	No variants detected	Negative
69	M	236.7	No variants detected	Negative
70	M	747.4	No variants detected	Negative
71	M	1492.4	No variants detected	Negative
72	M	1007.3	No variants detected	Negative
73	M	975.8	No variants detected	Negative
74	M	998.0	No variants detected	Negative
75	M	1018.6	No variants detected	Negative
76	M	618.8	No variants detected	Negative
77	M	874.0	No variants detected	Negative
78	M	983.3	No variants detected	Negative
79	M	1328.6	No variants detected	Negative
80	M	1415.7	No variants detected	Negative
81	M	1650.3	No variants detected	Negative
82	M	109.2	c.133-38C>A	One VUS. No second variant

(D) *ADA* gene

Case	Gender	Concentration (pmol/L)	Sequencing	Conclusion
		ADA 93		
83	F	3569.5	p.Ala215Thr	One VUS. No second variant
84	M	3486.6	No variants detected	Negative
85	F	1969.3	No variants detected	Negative
86	F	5278.0	No variants detected	Negative
87	F	3151.1	No variants detected	Negative
88	F	4763.6	No variants detected	Negative
89	F	4497.0	No variants detected	Negative
90	F	3740.5	No variants detected	Negative
91	F	2849.5	p.Arg76Trp	One VUS. No second variant
92	M	1856.4	No variants detected	Negative
93	M	4759.7	No variants detected	Negative
94	M	1652.3	p.Arg76Trp	One VUS. No second variant
95	F	3110.9	No variants detected	Negative
96	F	2251.0	No variants detected	Negative
97	F	3620.3	p.Arg76Trp	One VUS. No second variant
98	F	3439.0	No variants detected	Negative
99	F	4494.5	No variants detected	Negative
100	M	986.7	No variants detected	Negative
